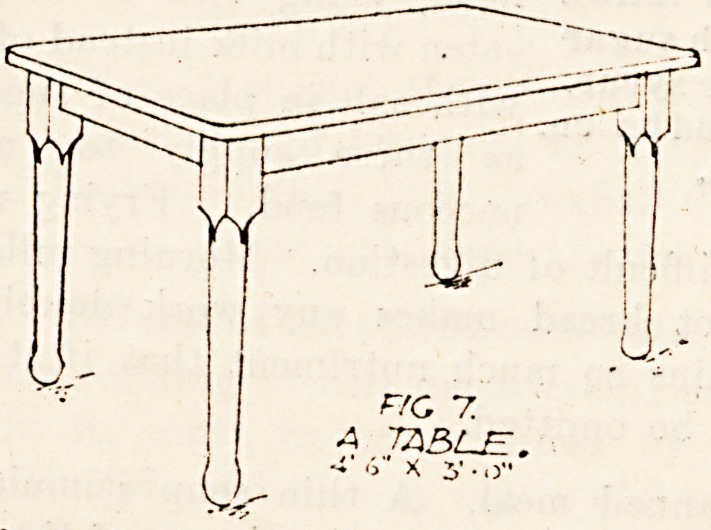# Practical Points

**Published:** 1911-11-25

**Authors:** 


### PRACTICAL POINTS.

(Criticism and Suggestions Invited.)

### Ward Furniture.

We publish this week illustrations of ward furniture as carried out at the new Royal Infirmary, Manchester.

Fig. 1 shows the type of ward locker which is made in American ash stained to a dark shade and

FIG 1. WARD LOCKER. 2' 6" WIDE 1' 2" DEEP.

FIG 2. A BED TABLE. 2' 0" x 1' 5"

varnished inside and out two coats. It has a lattice back and shelf, marble top, and marble medicine shelf with an iron rod surrounding it. The two

FIG 3 FOOD TROLLEY. 3' 3" x 1' 10"

hooks are for prescription sheet and temperature chart. Noiseless castors support the locker.

Fig. 2 illustrates a bed table, and one is provided for every two beds, 242 in all. This again is of ash stained and varnished to match the locker. When closed this article forms a convenient bed dining-table, and when opened on the ratchet arrangement can be adjusted to any angle to the

FIG 4. BOOK TROLLEY. 2' 0" x 1' 5"

comfort of the patient when reading. Steel brackets support it in position.

Fig. 3 shows a food trolley for the distribution of dinners, teas, etc., to the bed patients and for the collection of dishes thereafter. One is provided in each ward and it is fitted with zinc trays, the woodwork being of ash to match the other furniture, and it is supported on noiseless castors.

Fig. 4 is a trolley similar in design and material to the food trolley but without zinc trays. The

FIG 5. A WARD COUCH. 5' 9" x 2' 9"

purpose of this smaller trolley is to convey books and magazines round the ward.

Fig. 5 illustrates a ward couch which has a framework of ash stained and varnished, and upholstered in selected horsehair covered with best American cloth.

The ward walls have at their base the usual round or cavetto, and Fig. 6 shows a bed stop which is made to fit the curve at the junction of floor and wall. Its object is to prevent the bedstead from

FIG 6 BEDSTOP.

being pushed against and damaging the wall surface. Four hundred and eighty-three of these fitments are provided, one to each bed.

Fig. 7 illustrates the type of table. There are several sizes but all similar in design.

The servants' hall larger tables have six legs and measure nine feet by three. The top is of white deal covered with white oiled baize. The legs and framing are of ash. The smaller tables in servants'

FIG 7. A TABLE. 4' 6" x 3' 0"

hall are 4 feet 6 inches by 3 feet. Nurses' dining tables are in fumed oak to seat four or six persons in restaurant fashion. Twenty-four sisters' tables are 3 feet 10 inches by 3 feet 2 inches, and twenty-four ward tables 5 feet 6 inches by 3 feet 6 inches. The ward kitchen table is 4 feet 6 inches by 2 feet 6 inches, and there are likewise twenty-four of these.

## Figures and Tables

**Fig. 1. f1:**
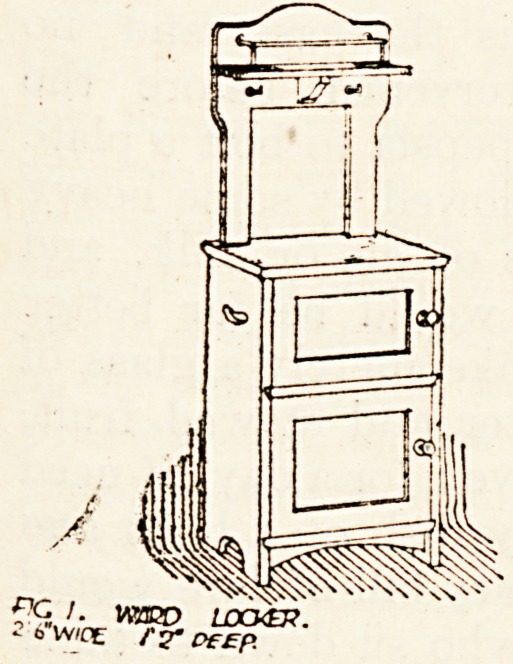


**Fig. 2. f2:**
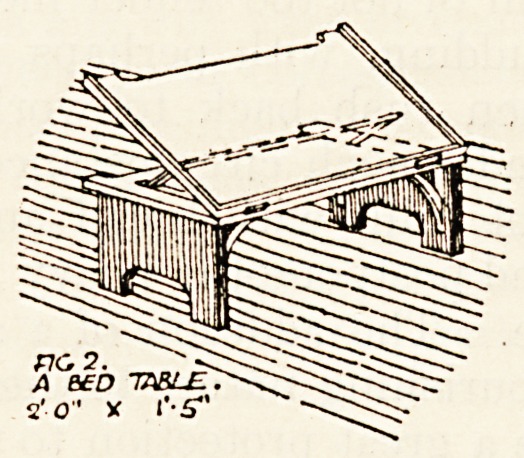


**Fig 3 f3:**
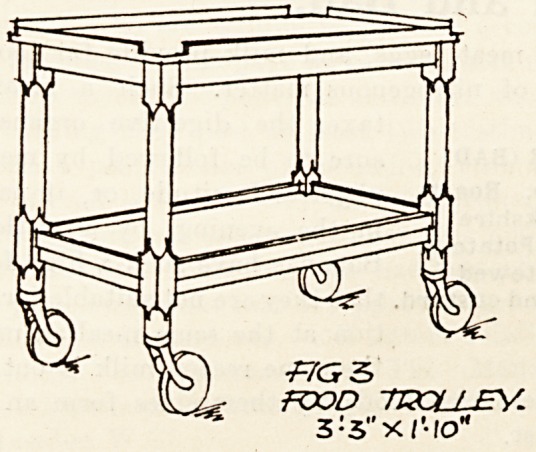


**Fig 4. f4:**
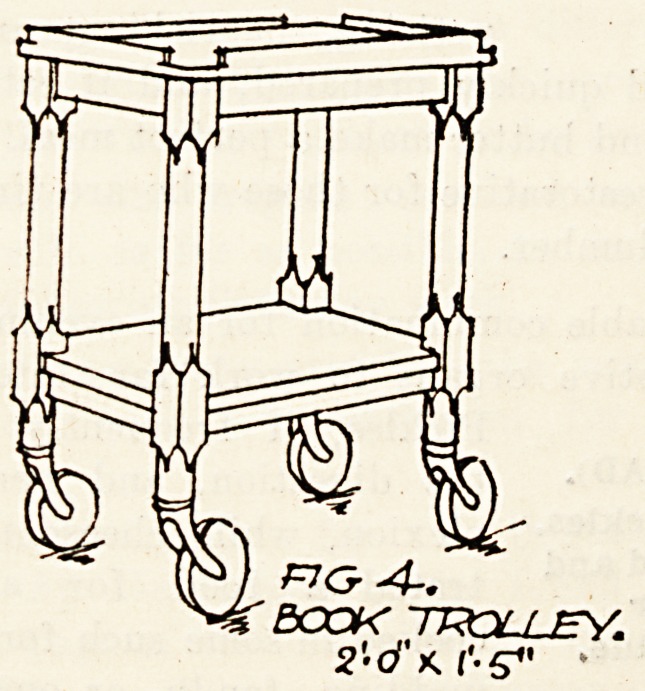


**Fig 5. f5:**
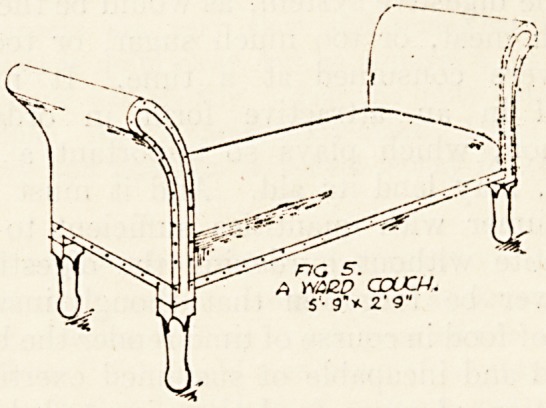


**Fig 6. f6:**
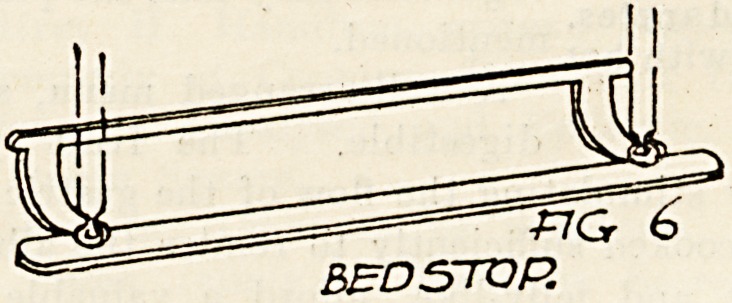


**Fig 7. f7:**